# Reconstruction clipping of ruptured anterior circulation aneurysms via supraorbital lateral keyhole approach

**DOI:** 10.1186/s41016-022-00272-6

**Published:** 2022-02-14

**Authors:** Yuzhang Wu, Yan Zhao, Shengping Yu, Fan Li, Shifei Cai, Chao Peng, Zhen Wang, Yifan Yang, Bangyue Wang, Xinyu Yang

**Affiliations:** Tianjin Medical University General Hospital, 154 Anshan Road, Heping District, Tianjin, China

**Keywords:** Intracranial aneurysm, Subarachnoid hemorrhage, Clipping

## Abstract

**Background:**

Intracranial aneurysm (IA) is a serious disease. Analyze and review the cases of anterior circulation ruptured IA by supraorbital lateral keyhole approach, and summarize the experiences of this approach.

**Methods:**

Retrospective analysis of 16 cases of ruptured anterior circulation IA in our department from January 2019 to June 2020, CT angiography (CTA) was performed before operation. Analyzing the IA’s parameters by 3D-CT reconstruction. The IA was clipped by supraorbital lateral keyhole approach combined with the 3D-skull reconstruction. Extraventricular drainage was performed before craniotomy. Intraoperative neurophysiological monitoring was performed during the operation. After operation, fluorescein angiography and vascular ultrasound were performed to check the clipping effect. Intracranial pressure monitor was performed postoperatively. CTA was reexamined one week after operation. The modified Rankin Scale (MRS) was performed 6 months after operation.

**Results:**

There were 7 males (43.8%) and 9 females (56.2%), and the average age is 52.31 ± 11.12 years old. Among them, 11 patients (68.8%) were anterior communicating artery aneurysms and 5 (31.2%) were middle cerebral artery aneurysms. All patients were out of hospital within 10 days without any death, without cerebral infarction, cerebrospinal fluid leakage and neurological impairments. About mRS score, after 6 months follow-up, 8 cases (50%) had 0 point, 4 cases (25%) had 1 point, and 4 cases (25%) had 2 points.

**Conclusions:**

For ruptured anterior circulation IA, the supraorbital lateral keyhole approach combined with ventricular drainage, intraoperative electrophysiological monitoring, and intraoperative vascular ultrasound is a safe and minimally invasive treatment. The application of reconstruction clipping can reconstruct the diameter of parent vessel and reduce the recurrence rate of IA.

## Background

When clipped the anterior circulation ruptured IA, the classical pterional approach has clear field of vision and large direction adjustment. Therefore, it has mature experience in this approach, but its huge wound and the obstacle of postoperative temporal muscle function are criticized [1]. In 1998, Perneczky first proposed the supraorbital lateral keyhole approach [2]. Due to the advantages of small wound, simple operation and short operation time [3], this approach is more and more used in the treatment of intracranial tumors and intracranial vascular diseases. However, the supraorbital lateral keyhole approach is relatively difficult compared with the pterional approach because of its small bone window and small intraoperative observation view. Especially in the case of subarachnoid hemorrhage (SAH) due to IA rupture, the intraoperative field of vision is narrower than that without rupture, which makes IA clipping more difficult.

For the operation of anterior communicating aneurysms and middle cerebral aneurysms, due to irregular IA body and wide IA necks, it is often necessary to reconstruct aneurysm clips, clip the large IA body and ensure the aneurysmal vessel unobstructed. In recent 2 years, we tried to clip the ruptured aneurysms of anterior communicating and middle cerebral artery through the supraorbital lateral keyhole approach, explored the reconstruction clipping of ruptured aneurysms through this approach, and summarized the experience.

## Method

### Inclusion criteria

(1) The operative approach was supraorbital lateral keyhole approach; (2) preoperative CT showed SAH, and there was a single aneurysm in the anterior circulation; (3) IA was clipped by reconstruction clipping; (4) posterior communicating artery aneurysms are not included because of the small IA carrying vessels. Multiple aneurysm clips may lead to distortion or even rupture of posterior communicating artery; (5) the case data are complete. Written informed consent for the publication of patient details was also obtained from the legal representative of all patients.

### Patient characteristics

From January 2019 to June 2020, 16 cases of anterior circulation ruptured IA reconstruction clipping through supraorbital lateral keyhole approach were counted, including 9 females and 7 males. The average age of the patients was 52.31 ± 11.12 years old. There were 11 ruptured anterior communicating aneurysms and 5 ruptured middle cerebral aneurysms. There were 3 cases of lobulated aneurysms, and the rest were cystic aneurysms.

### Preoperative preparation

After admission, the patients underwent head CT and CTA, 3D skull reconstruction and vascular reconstruction. According to IA position, size, and IA direction, the position and size of bone flap were determined. CTA can also observe the perforating vessels around IA, which can be avoided during operation and reduce the incidence of postoperative complications. The Fisher grade and Hunt-Hess grade were evaluated by head CT, and the morphology of lateral ventricle was observed to provide imaging guidance for ventricular puncture and drainages.

### Operation process

Firstly, ventricular puncture and drainage were performed: 9 cm behind the nasal root on the midline and 2.5 cm beside the midline. After catheterization, the intraventricular pressure was measured, the drainage tube was fixed, and the drainage bottle was closed.

#### Craniotomy via supraorbital lateral keyhole approach

**Fig. 1:**
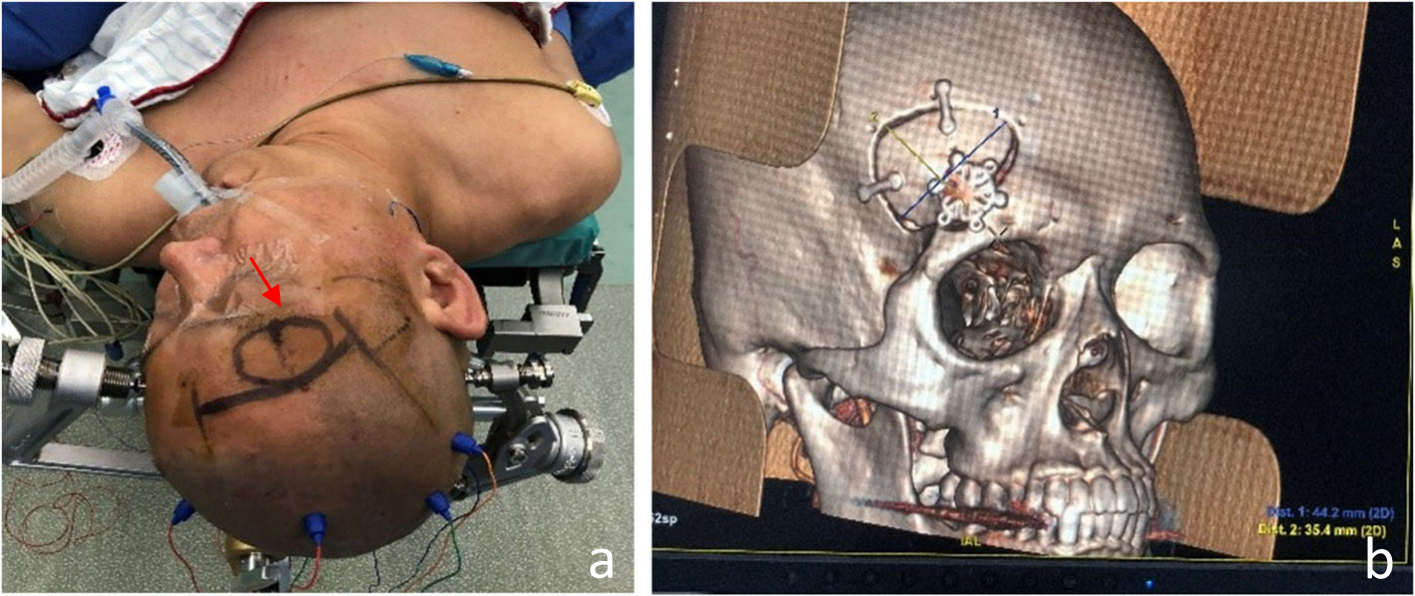
Preoperative preparation and postoperative bone flap display of ruptured anterior communicating aneurysm by reconstruction clipping via supraorbital lateral keyhole approach. **a** The patient had a ruptured anterior communicating artery aneurysm. The patient’s head was slightly left to the upper three-point skull frame, which was connected with electrophysiological monitoring. The arrow showed the approximate range of bone flap. **b** Postoperative 3D-CTA showed that the bone flap was well fixed

The patient’s head deviated slightly by 15–20° in supine position, connected with brain electrophysiological monitoring equipment, fixed head with three-point head frame, 4–5 cm arc incision at the front edge of hairline (Fig. 1), and opened the flap and temporal muscle to expose the skull. Drill a hole near the frontal horn process, and remove the bone flap. The size of the bone flap is about 3 × 3 cm. Grind the edge of the bone window, level the anterior skull base. Open the external ventricular drainage tube, slowly release about 50 ml of cerebrospinal fluid, open the dura mater, lift the frontal lobe, expose the optic nerve, open the carotid cistern and lateral fissure, and gradually expose the internal carotid artery, middle cerebral artery, and anterior cerebral artery, and select appropriate aneurysm clips to reconstruct IA. After clipping, fluorescence angiography and vascular ultrasound were performed to test the clipping effect. After clipping, the skull was closed routinely.

#### Reconstruction clipping

For IA with large, wide neck, lobulated, or other irregular shapes, we generally use reconstruction clipping. The method is to use multiple (≥ 2) aneurysm clips to clamp IA step by step, gradually reduce the aneurysm volume until the aneurysm neck is completely closed, and the line between the tip of the aneurysm clip is similar to the line between the intersection of the aneurysm neck and the IA carrying vessel, so as to repair the damaged vessel wall of the IA carrying vessel.

After operation, the drainage tube was removed, lumbar cistern puncture was performed, and the tube was placed for drainage.

## Results

From January 2019 to June 2020, we concluded 16 eligible patients with ruptured anterior circulation IA, contain 11 anterior communicating aneurysms (68.8%) and 5 middle cerebral aneurysms (31.3%). There were 7 males (43.8%) and 9 females (56.2%). The age range is 37–71 years old, and the average age is 52.31 ± 11.12 years old. Eight cases (50%) had hypertension before operation, and 2 cases had diabetes before operation (12.5%). Postoperative GCS score was good (≥ 13 points) in 14 cases (87.5%). Six months after discharge, the mRS score of 16 patients was 0 in 8 cases (50%), 1 in 4 cases (25%), and 2 in 4 cases (25%) (Table 1).

**Table 1:** Basic information of 16 patients

Total(%)			16(100%)
Sex(%)			
Male			7 (43.8%)
Female			9 (56.2%)
Age(%)			
< 40 years old			1 (6.3%)
40–60 years old			9 (56.2%)
> 60 years old			6 (37.5%)
Complications(%)			
Hypertension			8 (50%)
Diabetes			2 (12.5%)
Hemorrhagic stroke			1 (6.3%)
Chronic gastritis			1 (6.3%)
None			5 (31.3%)
Location IA(%)			
Anterior communicating artery			11(68.8%)
Middle cerebral artery			5 (31.3%)
Preoperative Hunt-Hess grade(%)			
Grade 2			9 (56.2%)
Grade 3			7 (43.8%)
Preoperative Fisher grade(%)			
Grade 2			8 (50%)
Grade 3			5 (31.3%)
Grade 4			3 (18.8%)
Preoperative WFNS(%)			
I			6 (37.5%)
II			7 (43.8%)
III			3 (18.8%)
Time from paroxysm to operation(%)			
< 24 h			12(75%)
24–48 h			3 (18.8%)
> 48 h			1 (6.3%)
Postoperative GCS score(%)			
15			6 (37.5%)
14			7 (43.8%)
13			1 (6.3%)
12			2 (12.5%)
mRs score 6 months after operation(%)			
0			8 (50%)
1			4 (25%)
2			4 (25%)

All the 16 patients had single ruptured IA, including 11 anterior communicating aneurysms (68.8%) and 5 middle cerebral aneurysms (31.2%). The width of aneurysm neck ranged from 2 to 10 mm, and the average width of aneurysm neck was 3.5 mm. Intraoperative reconstruction clipping used aneurysm clips ranging from 2 to 4. The operation time was 80–160 min, and the average operation time was 109.3 min (Table 2). No blood transfusion was performed in 16 patients. There was no secondary operation. Postoperative CTA showed that IA was completely clipped. There was no new postoperative cerebral infarction. Because of the small number of cases, no regression analysis was performed.

**Table 2:** Operation condition of 16 patients

Total(%)			16(100%)
Location of IA(%)			
Anterior communicating artery			11(68.8%)
Middle cerebral artery			5 (31.3%)
IA neck width(%)			
2 mm			5 (31.3%)
3 mm			6 (37.5%)
4 mm			3 (18.8%)
6 mm			1 (6.3%)
10 mm			1 (6.3%)
IA shape(%)			
Saccular			13(81.3%)
Lobulate			3 (18.8%)
Number of clips used(%)			
2			10 (62.5%)
3			5 (31.3%)
4			1 (6.3%)
Operation time(%)			
< 100 min			5 (31.3%)
100–120 min			8 (50%)
> 120 min			3 (18.8%)

## Case

### Case 1

**Fig. 2:**
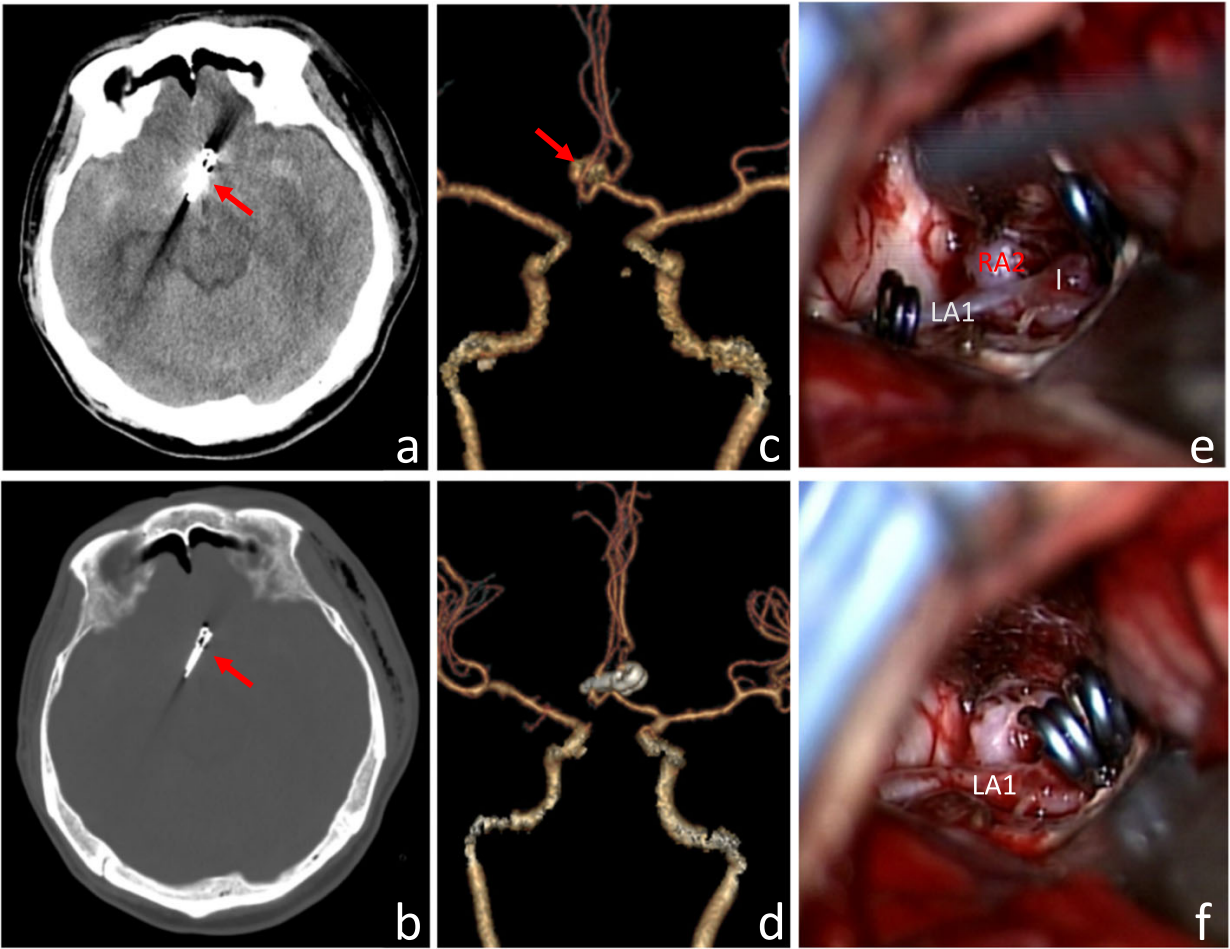
Preoperative and postoperative imaging examination and intraoperative findings of ruptured anterior communicating aneurysms with reconstruction clipping via supraorbital lateral keyhole approach. **a**, **b** On postoperative CT, there were 2 aneurysm clips(the arrow), and no postoperative bleeding and other complications were found. **c**, **d** Preoperative CTA: anterior communicating artery aneurysm with irregular shape. **e** Two temporary blocking clips blocked the IA carrying vessels; f: Two aneurysms clip clips the aneurysm. (LA1: A1 segment of left anterior cerebral artery; RA2: A2 segment of right anterior cerebral artery; IA: cerebral aneurysm)

The patient, a 56-year-old male, was hospitalized for “sudden dizziness for 11 h and aggravation with severe headache for 4 h.” Emergency CT prompt: SAH. CTA showed anterior communicating artery aneurysm. Hypertension in the past 5 years, up to 160/80 mmHg, poor blood pressure control. Hunt-Hess level 3. The initial pressure of extraventricular drainage was 15 mmHg. The cystic protrusion at the junction of A1–A2 on the left was found, and the IA top pointed to the right. Two aneurysm clips were used to reconstruct and clip the IA neck (models 760 and 750 of B. Braun Company, Germany) and performed postoperative lumbar cistern drainage. Intraoperative bleeding was 80 ml. Operation time was 90 min. Postoperative head CTA showed that the aneurysm was completely clipped and the perforating vessels were not damaged (Fig. 2). GCS at discharge was 15 points and mRS at 6 months after operation was 0 points.

### Case 2

**Fig. 3:**
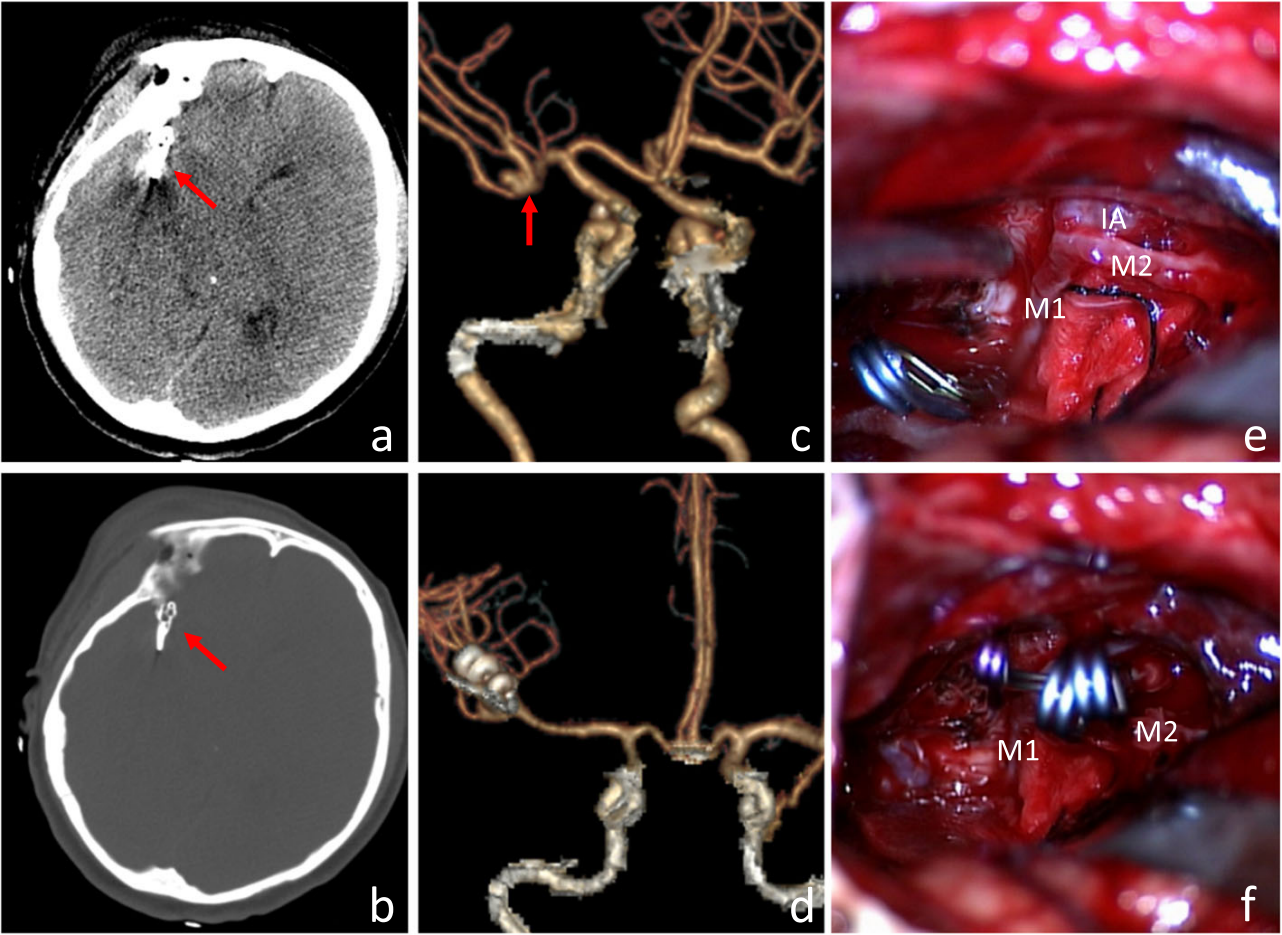
Preoperative and postoperative imaging examination and intraoperative findings of ruptured middle cerebral aneurysm with reconstruction clipping via supraorbital lateral keyhole approach. **a**, **b** The ruptured aneurysm was clipped with 3 aneurysm clips. The arrow is the aneurysm clip. **c** The arrow is the aneurysm at the M1M2 bifurcation of the right middle cerebral artery. **d** Postoperative CTA revascularization of the head showed that the aneurysm was completely clipped and the branch vessels were preserved. **e** The aneurysms were clearly exposed after temporary occlusion of the IA carrying vessels. **f** After clipping of three aneurysms, complete clipping was seen. (M1: M1 segment of middle cerebral artery; M2: M2 segment of middle cerebral artery; IA: cerebral aneurysm)

A 41-year-old female patient was admitted to the hospital because of “sudden headache with nausea and vomiting for 5 h.” CT: SAH. Head CTA: right middle cerebral aneurysm. Previous chronic gastritis for 4 years. Hunt-Hess grade 2. The initial pressure of extraventricular drainage was 7.5 mmHg. The right supraorbital lateral keyhole approach was used to find the wide necked aneurysm at the M1 bifurcation of the right middle cerebral artery. The top of the aneurysm pointed upward and outward, and aneurysm was clipped by 3 clips (German B-Braun Company, model 750, 760, mini 724).The lumbar cistern was performed after operation. The operation time was 120 min. Postoperative CT and CTA showed that the aneurysm was completely clipped and the perforating vessels were not damaged (Fig. 3). GCS 15 points at discharge and mRS 0 points 6 months after operation.

### Case 3

**Fig. 4:**
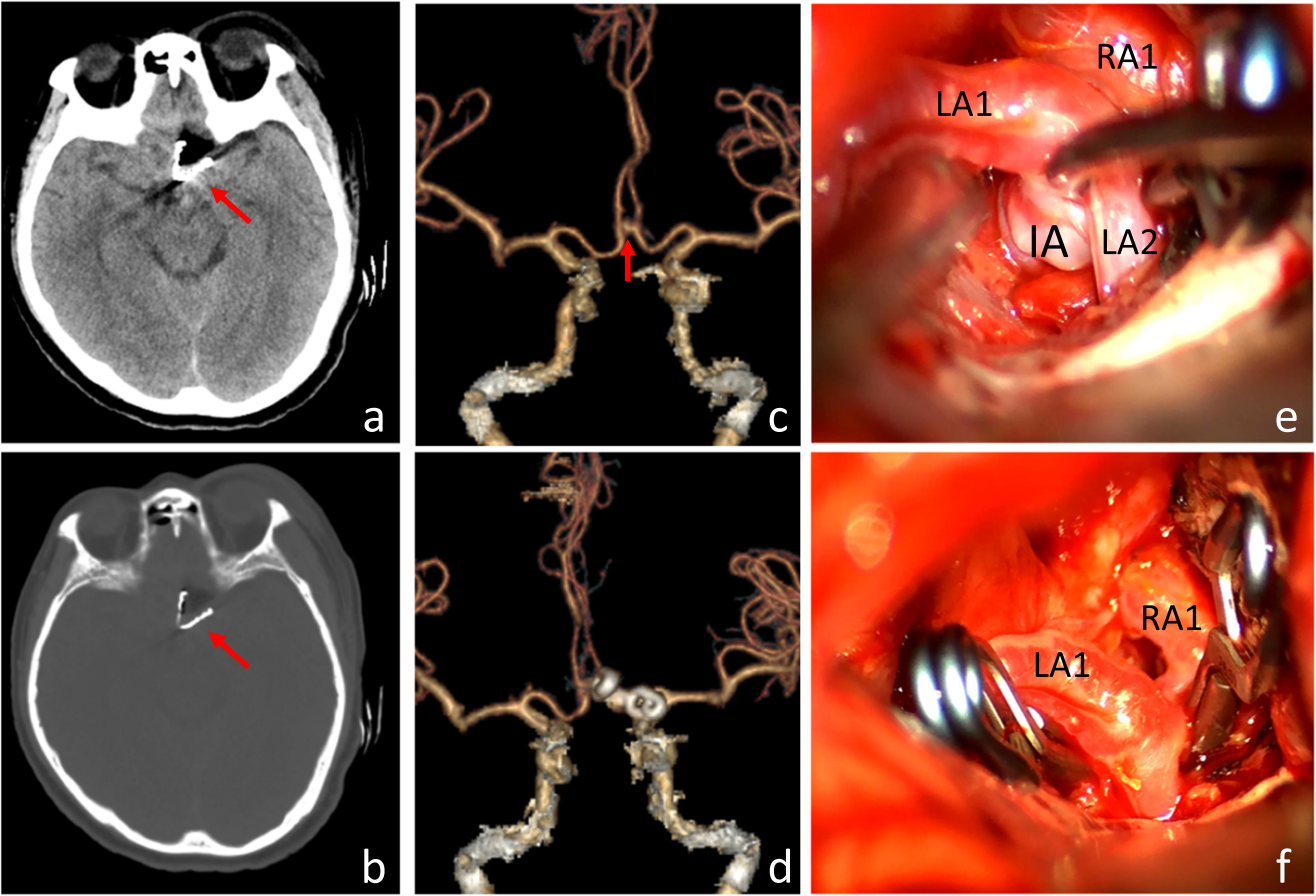
Preoperative and postoperative imaging and intraoperative findings of ruptured anterior communicating aneurysms with reconstruction clipping via supraorbital lateral keyhole approach. **a**, **b** The ruptured aneurysm of the patient's anterior communicating artery was shaped and clipped with 2 aneurysm clips. The arrow is the aneurysm clip. **c**, **d** CTA vascular reconstruction of the head showed that the aneurysm was completely clipped and the branch vessels were preserved. **e**, **f** Fully expose the IA neck during operation and clip it. (LA1: A1 segment of left anterior cerebral artery; LA2: A2 segment of left anterior cerebral artery; RA1: A1 segment of right anterior cerebral artery; IA: intracranial aneurysm)

A 64-year-old female patient was hospitalized for “sudden severe headache with unconsciousness for 4 days.” Head CT and CTA showed SAH and anterior communicating aneurysm. The past 15 years of hypertension. Hunt-Hess level 2. After intraventricular puncture and catheterization, the bilateral optic nerve, left internal carotid artery and A1 and A2 segments of bilateral anterior cerebral artery were gradually exposed through the left supraorbital lateral keyhole approach. The saccular aneurysm of anterior communicating artery was found, and the aneurysm neck pointed to the upper right. The aneurysm neck was fully exposed and clipped with two aneurysm clips (German B-Braun Company, model 740 and 742). Postoperative fluorescence angiography showed that the clipping was complete. Operation time was 160 min. Postoperative CT and CTA showed that the aneurysm was completely clipped (Fig. 4). GCS at discharge was 15 points and mRS at 6 months after operation was 0 points.

### Case 4

**Fig. 5:**
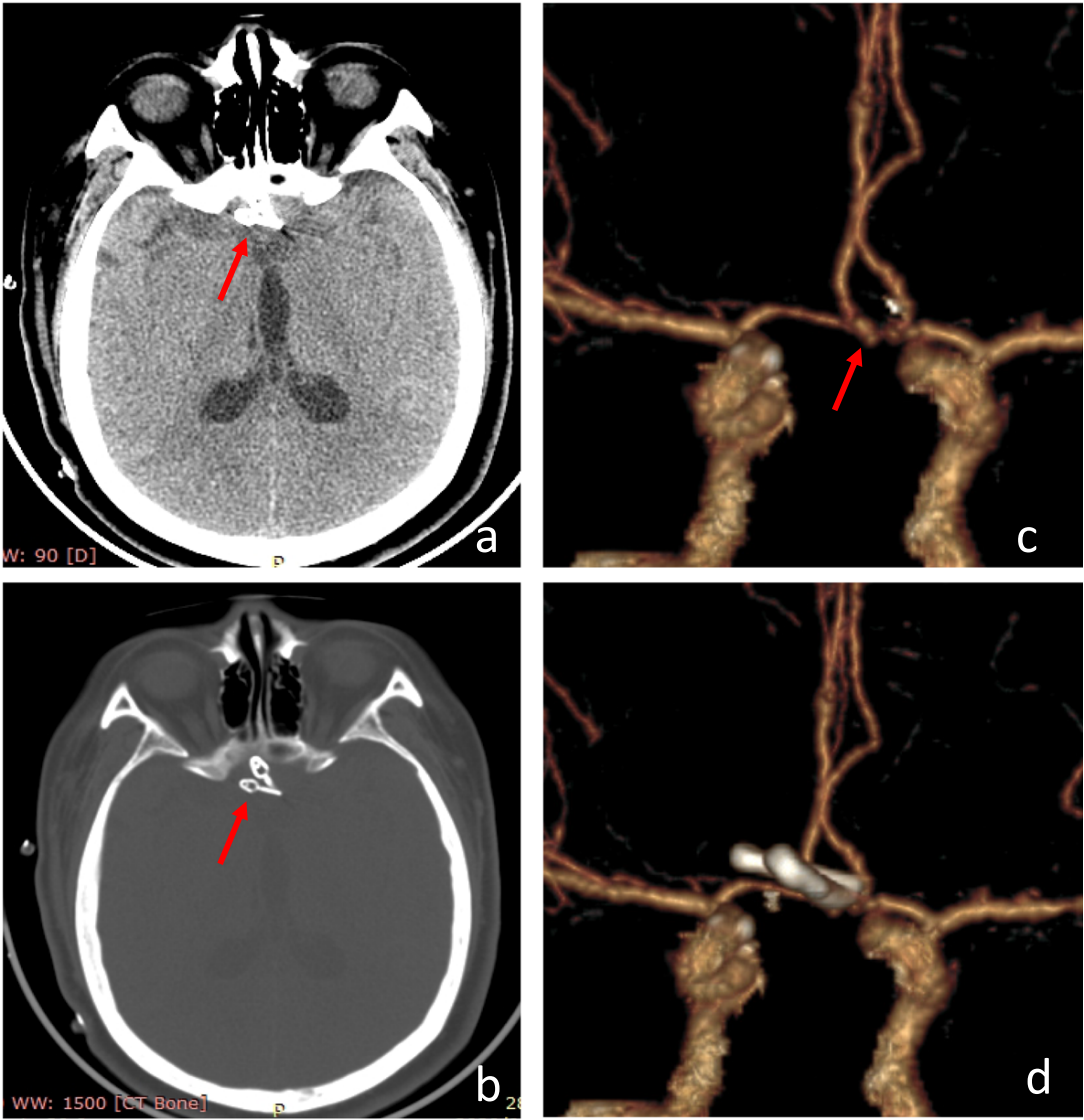
Preoperative and postoperative imaging examination and intraoperative findings of ruptured anterior communicating aneurysms with reconstruction clipping via supraorbital lateral keyhole approach. **a**, **b** On postoperative CT, there were 2 aneurysm clips at the arrow; **c** The shape of anterior communicating artery aneurysm at the arrow is irregular; **d** Postoperative reexamination showed that the aneurysm was completely clipped and other blood vessels were not damaged

The patient, a 49-year-old female, was hospitalized for “sudden dizziness, headache and consciousness disorder for 7 h.” Head CT and CTA showed SAH and anterior communicating aneurysm. She had hypertension for 7 years in the past, poor blood pressure control, and with the highest blood pressure of 200/100 mmHg. Hunt-Hess level 3, GCS 7 points (E: 2; V: 1; m: 3). After ventricular puncture and catheterization, the frontal lobe was lifted to expose the optic chiasmatic cistern and carotid cistern, the arachnoid was cut-off to see bilateral optic nerve and right internal carotid artery, the cystic process of anterior communicating artery was gradually explored and seen, and right A1, left A2, and right A2 were temporarily blocked. The aneurysm neck was continuously separated. It can be seen that the aneurysm is surrounded by the hematoma. During the removal of the hematoma, the aneurysm is torn and only a small piece of aneurysm neck tissue remains. Microsurgical suture of the adjacent residual aneurysm neck with a needle was done to establish the shape of the aneurysm. After that, two aneurysm clips (models FT740t and FT750t, German B-Braun Company) were used for reconstructing and clipping. Ultrasound showed that the clipping was complete and the IA carrying artery was unobstructed. Operation time was 130 min. Postoperative CT and CTA showed that the aneurysm was completely clipped (Fig. 5). GCS 14 points at discharge (E: 3; V: 5; m: 6).

## Discussion

Patients with ruptured aneurysm have severe headache, brain swelling and the risk of re-rupture and bleeding at any time. Therefore, surgery should be performed as early as possible, and an adequate and efficient preoperative preparation is very important. Head CT and head CTA three-dimensional reconstruction are the examination items that need to be completed as soon as possible before operation. Head CTA reconstruction can determine the size and position of bone flap. It can clearly show the relationship between IA and clinoid process and bone flap. The perforator of vessels was determined. More importantly, this examination can find the calcification of IA or IA carrying vessels and evaluate the probability of vasospasm or even vascular occlusion after temporary occlusion, so as to formulate a more comprehensive surgical plan. Due to the advantages of condition and CTA, the patients with ruptured IA were not routinely examined by DSA.

Small IA can use single clip to clip the IA neck, but for some IAs, such as wide neck, arteriosclerosis or calcification, irregular IA body, giant aneurysm, and single clip cannot be completely clipped. Therefore, there is a method of reconstruction clipping, which refers to clipping aneurysms with multiple clips while shaping the IA neck to restore the original diameter of the IA carrying vessel. Different from the traditional parallel placement of aneurysm clip, the direction of reconstruction clipping is more flexible. It aims to gradually reduce the volume of IA body, reduce its surface tension, retain the diameter of IA carrying vessels, and avoid damaging perforator vessels and surrounding brain tissue. After a large number of aneurysms were clipped, we found that lenticular artery and hypothalamic perforating artery were the most vulnerable arteries in middle cerebral aneurysms and anterior communicating aneurysms clipping operation. Therefore, special attention should be paid to whether these two arteries were unobstructed during operation. The “Picket Fence” clipping proposed by Davies and the “Mass Reduction” clipping proposed by Ririko also belong to reconstruction clipping [4, 5]. Using this method combined with intraoperative ultrasound can judge whether IA is completely clamped and whether perforating vessels are unobstructed, reduce postoperative complications, and prevent recurrence of IA.

The reconstruction clipping of IA reported in most literatures is carried out by using the pterional approach. The pterional approach can more fully observe the Willis ring; the intraoperative angle is large, which is convenient for IA construction clipping. Pterional approach must open the lateral fissure and lift the temporal lobe to see the vascular structure of the skull base, so it will inevitably damage the brain tissue. Studies have shown that intraoperative brain traction will damage the brain and even lead to permanent neurological deficit [6]. With the continuous development of interventional technology, compared with craniotomy clipping, more and more IA patients prefer coil embolization. Therefore, craniotomy as an operation that can cure cerebral aneurysm IA, the surgical incision must be reduced to achieve the purpose of minimally invasive, which makes neurosurgeons explore an approach with less damage.

Since Perneczky put forward the concept of keyhole approach [2], keyhole approach has been applied in more and more craniotomy operations with less trauma and good cosmetic effect. We believe that keyhole is not only the pursuit of small bone window, but the bone window that is suitable for the patient and has the least damage to the patient under sufficient preoperative evaluation (CTA, CT, etc.). A previous meta-analysis of this group proved that the supraorbital lateral keyhole approach is safe and effective, which can reduce the length of hospital stay and reduce the probability of postoperative infection [7]. Statistics show that there is no significant difference in mortality and postoperative complications between supraorbital lateral keyhole approach and pterional approach [8, 9], and this approach can avoid supraorbital nerve, frontal branch of facial nerve, superficial temporal artery and other structures and reduce iatrogenic injury. There were no complications such as frontal nerve injury and masticatory function impairment in 16 patients. Compared with the pterional approach, the supraorbital lateral keyhole approach has some disadvantages, such as narrow intraoperative field of vision and small operable range. These disadvantages can be improved by lengthening surgical instruments and accurate preoperative evaluation [1]. For exposure of IA, intracranial lesions on the brain surface need a bone window as large as the lesion itself to fully expose the lesions, while deep lesions can be exposed through a smaller and more limited approach [6]. Therefore, the “inverted funnel” exposure of anterior circulation aneurysms through a small bone window can be realized. The position of the bone flap is adjusted according to the position of IA. For example, the position of the bone flap of the M1 and M2 aneurysms of the middle cerebral artery is more lateral than that of the anterior communicating aneurysms, so as to fully expose the sphenoid ridge and lateral fissure.

Ruptured anterior circulation aneurysms are traditionally clipped by pterional approach. The treatment of superficial temporal artery and sphenoid crest during pterional approach is very time-consuming. Statistics show that the operation time of clipping IA through pterional approach is higher than that through supraorbital lateral keyhole approach [3, 9]. Long operation time and long-term exposure of brain tissue to non-physiological environments such as air, normal saline, and dressing will lead to damage to nerves and vessels on the brain surface [10]. Therefore, the supraorbital lateral keyhole approach with shorter operation time can reduce the probability of brain injury, especially for elderly patients. When using this approach, an extended clip holder was used, and the remaining instruments were not specially made. For the supraorbital lateral keyhole approach, large size and wide neck IA need larger incision and bone flap to apply multiple clips in different directions [11], so the bone flap produced by construction clipping IA is larger than that produced by simple clipping. Some literatures have pointed out that the larger frontal sinus is a contraindication of the supraorbital lateral keyhole approach [12], but the current bone wax and intraoperative disinfection make us no longer think that this is a contraindication of this approach. Because the frontal lobe is lifted during the operation, olfactory nerve injury may occur after the operation. According to the literature, the pterional approach clamping of ruptured IA has a high probability of olfactory nerve injury [13]. In a recent statistics, the probability of olfactory nerve injury was about 12% (23/188) by clamping ruptured IA through the supraorbital lateral keyhole approach [14]. The frontal lobe was carefully separated to protect the olfactory nerve. There was no olfactory injury in 16 patients. The use of intraoperative electrophysiological monitoring during ruptured IA clipping can reduce the incidence of complications [15]. We routinely used EEG monitoring, and motor nerve evoked potential monitoring during operation and began monitoring after temporarily blocking the IA carrying vessels to ensure the normal neural function of the blood supply area of the IA carrying vessels. Intraoperative aneurysm rupture is a common emergency. Careful and gentle separation of aneurysm neck, sharp separation of arachnoid, and slow traction of brain tissue can greatly reduce the risk of intraoperative ruptured IA. Many doctors worry that too small bone window may not be able to control bleeding in the event of intraoperative rupture of IA through the supraorbital lateral keyhole approach. This shows that the small bone window of this approach can send the instrument near IA for hemostasis. Controlling the proximal end of the IA carrying vessel is the consensus of clipping. It is equally important to fully expose and control the distal end of the IA carrying vessel, so as to “isolate” IA, minimize the blood flow in IA, and ensure the safety of operation. Hitoshi et al. believes that patients with poor Fisher grade (grade IV) before operation should not be treated by keyhole approach, and the risk of intraoperative IA re-rupture is very high [10]. Among 16 patients in this group, 3 (18.8%) were Fisher grade 4, and there was no re-rupture during operation. Therefore, whether the patients with severe condition and high preoperative Fisher grade are suitable for IA clipping via supraorbital lateral keyhole approach remains to be further studied. The 16 patients could take care of themselves after operation, and the scar of head incision was not obvious.

Ruptured IA will cause brain tissue swelling and increased intracranial pressure. In order to obtain sufficient operation space and reduce the traction of brain tissue, we routinely performed lateral ventricular puncture and drainage cerebrospinal fluid before operation. After the operation, open the drainage tube and slowly release about 50 ml of cerebrospinal fluid, which is enough to expose the carotid pool. It is reported in the literature that a patient with ruptured anterior communicating aneurysm underwent extraventricular drainage before operation. After drainage of 50 ml cerebrospinal fluid, brain stem herniation secondary occurred [14]. Rapid cerebrospinal fluid drainage may lead to accidental rebleeding of IA, so the drainage should be slow and mild [16]. At present, there are no guidelines for cerebrospinal fluid drainage volume and drainage speed in patients with ruptured IA. Therefore, it is necessary to release cerebrospinal fluid intermittently and in a small amount to prevent complications such as cerebral hernia caused by releasing cerebrospinal fluid under low intracranial pressure. If the patient has mental state change and unequal pupil after drainage, be alert to brain stem herniation secondary occurred. Postoperative cerebrospinal fluid drainage in patients with ruptured IA is considered to be a beneficial method for the disease [17]. We routinely removed the extraventricular drainage tube to prevent intracranial infection, performed lumbar cistern puncture and drainage, monitored the intracranial pressure with the intracranial pressure monitor, and removed the drainage tube one week later. Intracranial pressure monitor can reduce the pain caused by frequent lumbar puncture after operation, and record the intracranial pressure for easy analysis.

The limited working angle is the one of the major disadvantages of supraorbital lateral keyhole approach and limits the directions for observation. We think the anteriorly and superiorly projecting ruptured IA were suitable for this strategy. We suggest the IA neck ≥ 10 mm is not suitable for this approach, because the IA neck cannot be fully exposed.

The shape of IA is not limited, and the construction clipping can take care of it.

## Conclusions

For ruptured aneurysms of anterior circulation except the posterior communicating artery, we used CTA to reconstruct the skull and blood vessels before operation; we used extraventricular drainage, electrophysiological monitoring, vascular ultrasound, and microscopic angiography during operation; and we applied intracranial pressure monitoring after operation, so as to fully ensure the safety of patients during perioperative and operation. Combined with supraorbital lateral keyhole approach, it is a safe, minimally invasive and rapid treatment method. The intraoperative clipping of aneurysms can reconstruct the diameter of IA carrying vessels and greatly reduce the recurrence rate of aneurysms and ischemia of IA carrying vessels. There was no drainage after operation, the patient recovered quickly, and the patient was satisfied with the frontal wound. Therefore, this surgical scheme can be used as an option for the surgical treatment of ruptured anterior circulation aneurysms.

## Data Availability

The datasets used and analyzed during the current study are available from the corresponding author on reasonable request.

## Abbreviations

IA: Intracranial aneurysm
CTA: CT angiography
mRS: Modified Rankin Scale
SAH: Subarachnoid hemorrhage
